# Status Epilepticus in Chromosomal Disorders Associated with Epilepsy: A Systematic Review

**DOI:** 10.3390/genes14020299

**Published:** 2023-01-23

**Authors:** Luca Bergonzini, Jacopo Pruccoli, Ilaria Pettenuzzo, Rosa Pugliano, Luca Soliani, Anna Fetta, Duccio Maria Cordelli

**Affiliations:** 1 IRCCS Istituto delle Scienze Neurologiche di Bologna, U.O.C. Neuropsichiatria dell’età Pediatrica, 40138 Bologna, Italy; 2 Dipartimento di Scienze Mediche e Chirurgiche (DIMEC), Alma Mater Studiorum, Università di Bologna, 40138 Bologna, Italy

**Keywords:** Status Epilepticus, Angelman Syndrome, Ring 20 Syndrome, chromosomal disorders, epilepsy, anti-seizure medications

## Abstract

Status Epilepticus (SE) is a neurological emergency resulting from the failure of mechanisms of seizure termination or from the initiation of mechanisms that lead to prolonged seizures. The International League Against Epilepsy (ILAE) identified 13 chromosomal disorders associated with epilepsy (CDAE); data regarding SE occurrence in these patients is lacking. A systematic scoping review was conducted to outline current literature evidence about clinical features, treatments, and outcomes of SE in pediatric and adult patients with CDAE. A total of 373 studies were identified with the initial search; 65 of these were selected and regarded as SE in Angelman Syndrome (AS, *n* = 20), Ring 20 Syndrome (R20, *n* = 24), and other syndromes (*n* = 21). Non-convulsive status epilepticus (NCSE) is frequently observed in AS and R20. No specific, targeted therapies for SE in CDAE are available to date; anecdotal reports about SE treatment are described in the text, as well as various brief- and long-term outcomes. Further evidence is needed to precisely portray the clinical features, treatment options, and outcomes of SE in these patients.

## 1. Introduction

The International League Against Epilepsy (ILAE) defines Status Epilepticus (SE) as a condition resulting either from the failure of the mechanisms responsible for seizure termination or from the initiation of mechanisms that lead to abnormally prolonged seizures [1]. SE may lead to brief- and long-term neurological and systemic consequences; it represents a medical emergency and it requires immediate evaluation and management to prevent significant morbidity or mortality [1,2]. Indeed, the effects of prolonged seizures upon neurons may be deleterious, inducing neuronal death, neuronal injury, and alteration of neuronal networks; these effects mostly rely upon seizure types and duration, other than on the stage of brain development, with age-dependent distribution [1,3]. Overall, mortality may vary between pediatric and adult patients, ranging between 3% and 40% in convulsive SE (CSE) [4].

The incidence of SE varies by age, being highest in the neonatal period (135–150 events per 100,000 people) or in vulnerable populations with acute or chronic neurologic conditions [5].

Several genetic anomalies may be associated with seizures, epilepsy, and SE: the ILAE identifies 13 chromosomal disorders associated with epilepsy (CDAE) either presenting with distinct seizure and electroencephalographic (EEG) features or frequently seen in epilepsy populations (Table 1) [6].

Many of these abnormalities involve chromosomal regions that are likely to contain crucial genes for the development of seizures; others are associated with Central Nervous System (CNS) malformations and neurological alterations leading to seizures with a higher incidence than in the general population [7,8]. Over time, the phenotypical characterization of these patients allowed clinicians and researchers to identify typical clinical and EEG patterns for certain CDAE (i.e., Ring 20 chromosome syndrome, Miller–Dieker syndrome, 18q- syndrome, and Down syndrome); conversely, other conditions show no specific epilepsy features (i.e., Ring 14 syndrome and Klinefelter syndrome) [7]. However, comprehensive literature data regarding clinical features, treatments, and brief- or long-term outcomes of SE in pediatric and adult patients with CDAE is lacking. The aim of the present review is to gather evidence to identify possible specific characteristics of SE in these syndromes.

## 2. Materials and Methods

### 2.1. Protocol

This systematic review was performed in October 2022 by browsing the following databases: Medline (PubMed), Web of Science, and Clinicaltrials.gov (accessed on 1 October 2022). The research was enhanced by searching the most relevant websites of guidelines and clearinghouses and the most important textbook sites. The Preferred Reporting Items for Systematic Reviews and Meta-Analyses guidelines (PRISMA) flowchart was used to determine the transparent exclusion of published literature for defined reasons. Pertinent selected works were tabulated concerning the number and type of participants, study design, and main results.

Inclusion criteria:

(1) Date: published before October 2022.
(2) Population:
  - Infants, children, adolescents, and adults;
  - Patients with a diagnosis of syndromes due to chromosomal abnormalities frequently seen in people with epilepsy or with distinct seizure and EEG features, as listed by the ILAE, https://www.epilepsydiagnosis.org/aetiology/chromosomal-abnormalities-overview.html (accessed on 1 October 2022);
  - Patients who experienced at least one episode of SE during their lifetime.
(3) Language: English.
(4) Study Design: RCT, cohort studies, cross-sectional studies, retrospective studies, case reports.
(5) Outcomes: reporting at least one between:
  - SE clinical features (semeiology, symptoms/signs, duration before diagnosis);
  - SE treatment;
  - SE outcome (response to treatment and recovery).

Exclusion criteria:

(1) Population: other than humans;
(2) Language: other than English;
(3) Study design: descriptive studies, reviews, protocols;
(4) Missing outcomes of interest.

Given the scoping nature of this systematic review, as well as the scarce evidence existing in the field, both controlled and uncontrolled studies were included.

Search strings (Box 1) included all syndromes and related synonyms gathered under the same rare disease nomenclature on OrphaNet [9].

Box 1 **Search strings**

((Angelman syndrome) AND (Status epilepticus) AND (english[Language])); ((15q13.3 syndrome) OR (15q13.3 microdeletion syndrome) AND (status epilepticus) AND (english[Language])); ((Chromosome 18 deletion syndrome) OR (18q- Syndrome) OR (Chromosome 18 Long Arm Deletion Syndrome) OR (Chromosome 18, Monosomy 18Q) OR (Del(18q) Syndrome) OR (Monosomy 18q Syndrome) OR (Chromosome 18q- Syndrome) OR (Chromosome 18q Deletion Syndrome) OR (Chromosome 18, monosomy 18Q) OR (Monosomy 18q, deletion 18q) OR (Monosomy 18q syndrome) OR (Chromosome 18q syndrome) OR (18q syndrome) AND (status epilepticus) AND (english[Language])); ((Inverted duplicated chromosome 15 syndrome) OR (inv-dup15) OR (Duplication/inversion 15q11) OR (Isodicentric chromosome 15 syndrome) OR (Non-distal tetrasomy 15q) OR (Non-telomeric tetrasomy 15q) AND (status epilepticus) AND (english[Language])); ((Del(1)(p36)) OR (Deletion 1p36) OR (Deletion 1pter) OR (Monosomy 1p36) OR (Monosomy 1pter) AND (status epilepticus) AND (english[Language])); ((Pallister-Killian syndrome) OR (Isochromosome 12p syndrome) OR (Isochromosome 12p mosaicism) AND (status epilepticus) AND (english[Language])); ((Trisomy 21) OR (Down syndrome) AND (status epilepticus) AND (english[Language])); ((47,XXY syndrome) OR (klinefelter syndrome) AND (status epilepticus) AND (english[Language])); ((Miller-Dieker syndrome) OR (Lissencephaly due to 17p13.3 deletion) OR (Monosomy 17p13.3) OR (Telomeric deletion 17p) AND (status epilepticus) AND (english[Language])); ((Ring chromosome 14 syndrome) OR (Ring chromosome 14) OR (Ring 14) AND (status epilepticus) AND (english[Language])); ((Ring chromosome 20 syndrome) OR (Ring 20) OR (Ring chromosome 20) AND (status epilepticus) AND (english[Language])); (Wolf-Hirschhorn syndrome) OR (4p- syndrome) OR (Distal deletion 4p) OR (Distal monosomy 4p) OR (Telomeric deletion 4p) AND (status epilepticus) AND (english[Language])); ((Partial trisomy/tetrasomy of the short arm of chromosome 12) OR (Partial duplication/triplication of chromosome 12p) OR (Partial duplication/triplication of the short arm of chromosome 12) OR (Partial trisomy/tetrasomy of chromosome 12p) AND (status epilepticus) AND (english[Language]))

**Figure 1:**
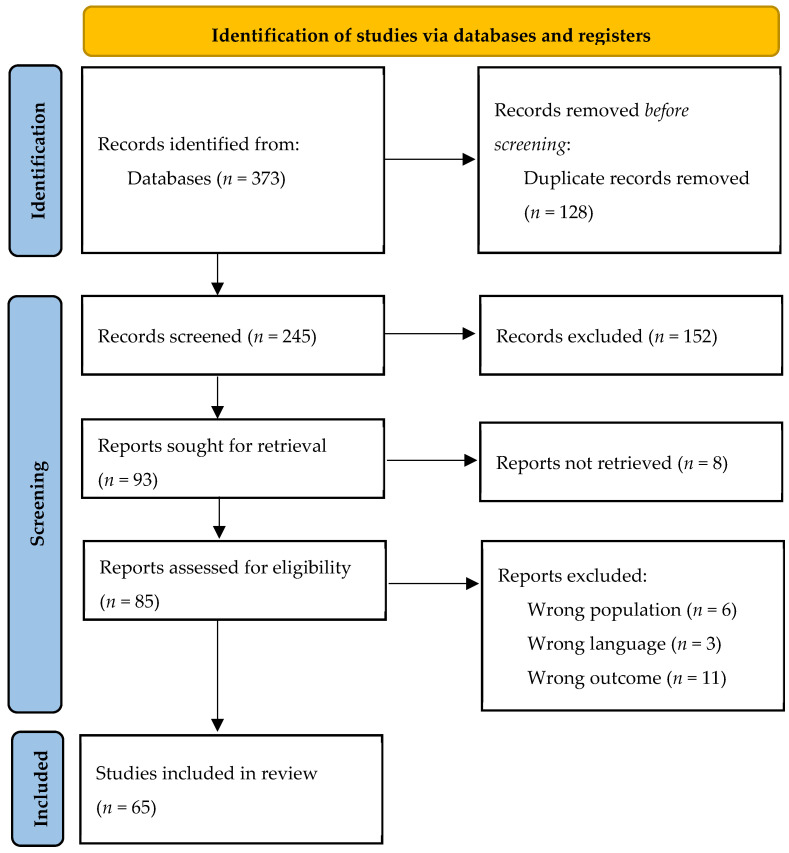
PRISMA flow chart.

A total of 373 original articles were identified. These were screened for appropriateness by two independent reviewers, according to inclusion criteria; papers not in English were included in case the desired outcomes were reported in the English abstract. Disagreements among reviewers were resolved through a mediator in the screening phase and the full texts of retained records were sought for retrieval by two independent reviewers. The eligible papers were included in the systematic review and categorized on an informatic spreadsheet. Risk-of-bias assessment was not systematically performed given the high prevalence of case reports and case series among the included papers; all the pertinent studies were included in order to provide a more comprehensive overview, in light of the lack of systematic reviews in this field. Sixty-five studies were included in the final analysis. The flow chart of the study is reported in Figure 1.

### 2.2. Statistical Analysis

The included studies mainly encompassed case reports and case series describing several outcomes of interest in a small number of patients; therefore, no meta-analysis was performed. Data were presented on a descriptive basis, and results were provided for the overall group of research.

## 3. Results

### 3.1. Angelman Syndrome

Twenty papers regarding the occurrence of SE in AS were included; the studies provide details on the history of epilepsy, SE semiology, recurrence, clinical signs, triggers, EEG pattern, duration treatments, and outcome (Table 2); one of them is a case-control study [10]. Overall, 150 patients with SE and AS were described.

#### 3.1.1. Epilepsy History

Information about epilepsy history was provided for 66 patients and most of them (*n* = 58) had seizures or epilepsy before SE; paroxysmal events with subtle motor features were retrospectively recognized as short atypical absences with eye-blinking in one patient [22].

#### 3.1.2. Clinical Features of Status Epilepticus in Angelman Syndrome

With regards to SE semiology, the majority of events were classified as NCSE; CSE, myoclonic status epilepticus (MSE), or electrical status epilepticus in sleep (ESES) were observed, as well. The youngest patient with NCSE and AS described was an 8-month-old boy, who experienced psychomotor retardation and NCSE recurrence over time, with a poor developmental outcome [19]. Half of the reports included described SE recurrence over time in patients with AS [10,12,15,17,18,19,21,25,26,28]. Table 3 gathers suggestive clinical symptoms or signs of NCSE among this population.

Possible SE triggers in patients with AS were suggested: (1) anti-seizure medications (ASMs), such as carbamazepine (CBZ) [10,13] (NCSE or MSE), oxcarbazepine (OXC), and vigabatrin (VGB) [10] (MSE); (2) fever and infections [10,24,26]; (3) sleep deprivation [26]. The EEG pattern of NCSE in patients with AS showed continuous, diffuse, high-voltage 1.5–3 Hz slow spike-wave (SW) complexes, with occasional frontal predominance [11,12,13,14,17,19,22,24,26,29]. Moreover, Uemura et al. observed the EEG pattern evolution in AS cases over a 30-year follow-up: every patient showing diffuse SW lasting for more than 20 s (“continuous diffuse SW pattern”) except one had NCSE (atypical absence status) during the follow-up period. The overall duration of NCSE episodes ranged between days or weeks, often as a consequence of significant diagnostic delays due to subtle or absent clinical signs [13,24,26,28].

#### 3.1.3. Treatment of Status Epilepticus in Angelman Syndrome

With regards to the treatment of SE in AS, the majority of reports referred to NCSE and described the use of benzodiazepines (BZDs) (clonazepam—CZP, diazepam—DZP, and midazolam—MDZ) as first-line therapy in most patients [11,12,13,19,24,26,29]. Among ASMs, valproate (VPA), and levetiracetam (LEV) were administered either as first or second-line treatment and frequently led to SE resolution [13,19,22,24]. Other authors described the efficacy of adrenocorticotropic hormone (ACTH) in two preschoolers with AS and refractory NCSE. Melikishvili et al. reported the case of two sisters harboring a novel *UBE3A* variant (c.2365del) who experienced refractory NCSE; the condition was treated with Ketogenic Diet (KD) (4:1 ratio), which proved effective within 2–3 days after initiation and before ketone bodies were detected in the urine [29]. Viani and Nicita observed the efficacy of VPA (as monotherapy or with Clobazam-CLB) or LEV, respectively, in the treatment of MSE [13,24]. Matsumoto et al. described the administration of Thyrotropin Releasing Hormone (TRH) to a 10-year-old boy with NCSE (atypical absence status) that did not resolve with DZP [12].

#### 3.1.4. Outcome of Status Epilepticus in Angelman Syndrome

Several authors have described SE outcomes in this population. Viani and Matsumoto observed low or absent NCSE recurrence in three patients as they turned 6, 7, and 15, respectively [13]. Melikishvili reported no recurrence of NCSE over a 5-year follow-up in two patients with SE onset at the age of 4 and 5 [29]. Ohtsuka et al. highlighted the onset of developmental delay roughly coincided with the appearance of NCSE in the series of cases they described; particularly, one patient with repetitive episodes of NCSE presented a very poor developmental outcome [19]. Bindels et al. observed a delay of days or weeks in the correct diagnosis of NCSE implying a long time to recover to the pre-NCSE level of functioning [28]. MSE occurred also in adulthood in the two patients described by Laan et al. [15].

### 3.2. Ring 20 Syndrome

Twenty-four studies about SE in R20 were included; the studies provide details on the history of epilepsy and use of ASMs, SE features (semiology, recurrence, clinical signs, triggers, duration, treatments, and outcomes (Table 4); two of them were case-control studies [30,31]. Overall, 93 patients with R20 and SE were described.

#### 3.2.1. Epilepsy History

Information about epilepsy history was provided for 67 patients: most of them (*n* = 62) experienced seizures or epilepsy before SE.

#### 3.2.2. Features of Status Epilepticus in Ring 20 Syndrome

With regards to SE semiology, most patients experienced NCSE (*n* = 66); exclusive CSE episodes or NCSE with CSE episodes occurred in a minority (*n* = 3 and 4, respectively); no information about SE semiology was available in 25% of patients (*n* = 24). Age at first SE ranged between 2 and 54 years of age. The frequency of SE over time ranged from single episodes to several per day [32,37,45,46]; overall, NCSE recurrence in patients with R20 regarded half the patients (*n* = 47). The clinical description of SE was not available for a total of forty patients. Table 5 lists clinical symptoms or signs of NCSE described in patients with R20. Further details about CSE semiology were not provided.

Potential triggers leading to NCSE were reported: (1) hyperventilation [32]; (2) CBZ [44]; (3) verbal stimuli—such as trying to respond to commands and questions [38]; (4) psychological fatigue [48], concentration [51] or emotional stress [34]; (5) hot bath [48]; (6) playing video games [32].

Information about EEG recording during NCSE was available for 55 patients with R20; the ictal records highlighted (1) slow waves and spikes or SW complexes predominantly over the frontal regions (*n* = 45) [30,32,34,36,45,46,48,51]; (2) generalized rhythmic slow waves or SW (*n* = 10) [36,37,38,40,42,45,49]. The overall duration of SE episodes spanned from 20 min to several days; Wechapinan reported one episode of NCSE lasting one month [46].

#### 3.2.3. Treatment of Status Epilepticus in Ring 20 Syndrome

Data about SE treatment in R20 were described in 19 patients, mostly regarding NCSE (*n* = 16). Two patients improved after administration of BZD (intravenous DZP [32] and CLZ [37]; MDZ along with LEV, CLB, zonisamide (ZNS), and lamotrigine (LTG) were necessary in one case [46].

Inoue et al. reported one patient responding to an intravenous infusion of lidocaine [32]. Kobayashi described a patient who responded to intravenous administration of phenobarbital (PB) [33]. Sodium thiopental, after administration of MDZ, phenytoin (PHT), and high-dose PB resolved one tonic CSE [49]. In two cases, lacosamide (LCS) led to clinical and EEG improvement [47,51]. LTG alone and as add-on therapy with VPA led to a slight improvement, according to Vignoli et al. [48].

Borkovic et al. reported improvement in seizure frequency and NCSE occurrence in two patients using LTG and Vagus Nerve Stimulation; one of them also needed ethosuximide as an add-on [53]. One case report suggested a good response to intravenous methylprednisolone [52]. On the other hand, intravenous PHT and VPA were administered in two patients with no NCSE resolution [38].

#### 3.2.4. Outcome of Status Epilepticus in Ring 20 Syndrome

Scarce information was found concerning brief- or long-term outcomes of SE in R20. Jacobs et al. reported a single case of death in the aftermath of prolonged, refractory NCSE occurring in one young boy; death was due to cardiovascular collapse when therapeutic measures were withheld [43]. Inoue et al.’s long-term follow-up (6 to 24 years after NCSE) of six patients described the absence of structural lesions or evident neurological involvement in the aftermath of SE [32].

### 3.3. Wolf–Hirschhorn Syndrome

Eight papers about SE in WHS were included (Table 6). Sixty-seven patients with SE and WHS were identified. Four patients did not have positive seizure history before SE onset. Patients with WHS almost exclusively experienced events with prominent motor features; in two cases, NCSE appeared after the cessation of motor SE [54], and one patient had prolonged atypical absence [55]. SE semiology was not described in 14 cases. Table 7 describes SE semiology in patients with WHS.

The age at onset of CSE was reported to range from 3 months to 3 years. Ten patients had recurrent SE, occurring with monthly to yearly frequency [20,54,55,56,57], while three patients presented only one episode of SE [54]. Suggested triggers for SE in patients with WHS were fever (in pediatric patients) [54,57,58], tiredness or excitement [58], and hot baths [54].

Three articles reported ictal EEG: Kagitani et al. observed left hemispheric spikes and slow-wave bursts with right deltoid muscle contraction during hemiclonic SE [54]. Caraballo et al. described rhythmic theta-delta activity and occasional SW complexes over the posterior regions [20]. Kanazawa et al. recorded marked asymmetry characterized by two Hz rhythmic slow waves and irregular spikes or slow waves localized in the left occipital area and low amplitude EEG in the right hemisphere (during hemiconvulsive SE) [56].

SE lasted more than 30 min in 13 out of 66 patients [54,57,60].

SE in WHS was treated with PB in four patients [54,55,57,60], with no improvement in one case. ZNS controlled hemiconvulsive SE in one case [55]. Effective administration of intravenous BZD together with PHT (4) or alone was reported in nine cases [54]; lidocaine, thiamylal sodium, and thiopental were reported in one case each [54]. Intubation was required because of respiratory insufficiency due to ASMs in three patients [54]. Six patients had a good response to bromide in preventing or controlling SE [54,59]. Pharmacological treatment was not reported for fifty-four patients [20,58,59].

Permanent paresis after SE was described in two patients and one patient had a fatal outcome due to pulmonary edema during SE [54].

### 3.4. Down Syndrome

Two studies about SE in DS were included. Four patients with SE were identified. SE occurred as the first seizure in two patients. As for semiology, three adult patients out of four presented an episode of MSE. SE semiology was not described in a 2-year-old boy presenting SE during measles infection, who developed extensive multiple cerebral infarctions (Moyamoya-like) after SE [62]. Only one ictal EEG pattern was reported and consisted of SW and polySW over temporal bilateral slow activity with associated eyelids myoclonia [61]. SE was resolved with intravenous VPA administration in two cases [61].

### 3.5. Ring 14 Syndrome

Two studies about SE in patients with R14 syndrome were included and 13 patients with SE were identified.

Four patients presented NCSE, 10 experienced SE with prominent motor features (with generalized tonic–clonic, unilateral clonic, tonic, and myoclonic–tonic features), and three had SE without any further characterization [63,64]. Clinical signs of NCSE included decreased alertness, behavioral modification, mild confusion, and reduced motor repertoire along with alternating gestural automatisms [64].

An EEG pattern of bilateral frontal high voltage continuous rhythmic delta activity was associated with NCSE [64]. No ictal EEG about CSE was described.

Treatment of NCSE required intravenous PHT, as intravenous lorazepam (LZP) resulted in a poor response in one patient [64]. CSE responded to intravenous LZP after DZP injection in one case [64], while high-dosage administration of barbiturates and PHT was reported to be efficient, as an initial treatment with BZDs often failed to obtain control in nine cases [63].

### 3.6. Other CDAE

With regard to the remaining CDAE, six studies about the occurrence of SE were reported in the literature.

Two studies about SE in patients with deletion of 15q13.3 were included. Four patients with SE were identified: two of them developed ESES [65,66], two experienced absence SE and one had focal to bilateral CSE [65]. Age at SE onset ranged between 3 and 7 years of age. The only described EEG pattern regarded the ESES episode, showing a significant increase in unilateral or bilateral SW complexes during sleep, with an SW index over 75% [66]. Neither the administered treatments nor the outcomes were reported.

One case report of SE in Del18q syndrome has been published [67]: the patient developed one episode of SE with prominent motor features on a previous personal history of generalized tonic–clonic epilepsy. The semiology of CSE was characterized by alternative repetition of horizontal nystagmus to the right and clonic convulsion of the right extremities every several minutes. EEG recording during SE showed continuous high voltage slow waves superimposed by spikes and polyspikes which developed into irregular spike discharges in the left occipital region at the end of the status. Neither treatment nor outcome was reported.

One study about SE in Del1p36 syndrome was included [68]. The authors described four patients (three males and one female) with the syndrome; all of them developed SE between 11 months and 4 years of age. All of them had positive epilepsy history; recurrent prolonged clusters of events associated with apnea and lasting up to 48 hours were reported in three patients, with monthly frequency. No description of SE semiology or therapy was included, but all patients required pediatric intensive unit care admissions.

InvDup15 syndrome was reported to be associated with SE in one included case-control study comparing living patients to SUDEP cases, with this CDAE [69]; seven patients (age range: 10–26 years old) with a previous history of drug-refractory epilepsy developed SE. No SE semeiology or specific treatment of SE was reported. Three of them had fatal outcomes due to SE.

One case report about SE in Klinefelter syndrome emerged from our systematic research [70]: the patient had maternal nondisjunction and uniparental disomy of the X chromosome together with interstitial Xp22.31 deletion of both X chromosomes. He developed SE at 7 months of age, with pallor, duskiness, head and eye deviation, and rhythmic jerking of his extremities. SE was triggered by infection, lasted two days, and showed refractoriness to treatment; the patient needed assistance in the Intensive Care Unit with intubation, and resolution occurred with the administration of PB, fosphenytoin, and LEV. The clinical course led to a stroke, followed by severe brain atrophy and developmental regression till death due to respiratory complications at 3 years of age.

No studies regarding SE in Pallister–Killian syndrome, Miller–Dieker syndrome, and Trisomy 12p were included in the present review.

Although not included among CDAE, the literature research highlighted three studies about SE in Ring 17 Syndrome (R17), which were included as secondary findings [71,72,73]. Three patients with NCSE were described. NCSE manifested with impaired awareness [72,73], and right-hand paresthesia [73]. In one case no clinical symptoms were identified except dubious rare brief head drop [72]. Ictal EEG patterns reported sub-continuous diffuse slow SW complexes over a background of slow activity in all cases. All of them developed epilepsy in the first decade and SE was reported between 11 and 28 years old. NCSE lasted about three days in one case [73]. One case was treatment-resistant [72], while improvement with BZD was reported in one case [73].

## 4. Discussion

Overall, the available evidence from studies published over the years suggests that CDAE may be associated with the occurrence of SE. The present literature review gathers current information about the occurrence of SE in patients with 10 CDAE. Secondary findings about SE in R17 are reported, even though the syndrome is not yet listed among CDAE with distinct seizure and EEG features or frequent occurrences in epilepsy populations by the ILAE [6]. Great heterogeneity in reporting the outcomes of interest emerges from the literature research; therefore, no proper inference can be made from the collected data. Most of the gathered data regard AS and R20 syndrome; therefore, a deeper analysis of the outcomes of interest was possible with regard to these syndromes.

### 4.1. Status Epilepticus in Angelman Syndrome

No cumulative data about prevalence or incidence can be collected due to the high predominance of case reports or case series over the literature. Valente et al. describe the occurrence of SE in 84% of patients with epilepsy and AS from their series, probably due to the study setting, in which patients went through prolonged video-EEG monitoring [10]. On the other side, Uemura et al. describe the occurrence of SE in 47.8% of 23 patients with AS enrolled in a long-term follow-up study on epileptic seizures and EEG findings in the syndrome [18]. Several authors have hypothesized possible genotype-phenotype correlations concerning SE semiology and genetic mechanisms underlying AS; however, most of the studies included in the present review did not provide information in this field [17]. NCSE and CSE occurred in 19% and 5%, respectively, of a large cohort of patients with AS [28]. Moreover, every attempt to evaluate the incidence of these events relies upon their identification by clinicians or caregivers. Indeed, most patients with AS experienced singular or recurrent NCSE episodes, which often represent a diagnostic challenge due to their subtle or absent clinical features. Among patients with AS, a typical behavioral pattern with a happy demeanor, prominent smiling, poorly specific laughing, general exuberance, hypermotor behavior, and stereotypies is described [74]. The interruption of this typical pattern, with the appearance of motor clumsiness, drooling of saliva, dulling of responsiveness and somnolence, as well as a lower frequency of tonic–clonic and atonic seizures in those regularly experiencing them, may suggest the occurrence of NCSE. Positive or negative signs pointing to NCSE reported over the literature are listed in Table 2. Poor evidence was collected in terms of SE trigger factors; sporadic reports about the role of CBZ, OXC, and VGB as MSE facilitators in children or adults with AS were found [10,13]; fever-induced SE was reported, as well.

With regards to SE treatment in AS, data are consistent with international guidelines, and BZDs and ASMs were administered as first- and second-line therapy; no specific, targeted therapies are described in the literature. Occurrence of refractory NCSE was observed, but no clear-cut risk factors for refractoriness (i.e., diagnostic delay) were identified. The efficacy of neuropeptides (ACTH), anesthetics, steroids, or KD to treat refractory SE has been described anecdotally [12,19,26,29]. Another neuropeptide, TRH, was successfully administered in 1992 by Matsumoto et al. to treat a 10-year-old boy with NCSE (atypical absence status), which did not resolve with DZP [12]. TRH use has been reported in West and Lennox–Gastaut syndrome, as well as early infantile epileptic encephalopathies unresponsive to ACTH and ASMs. Scarce evidence about its role in SE is available; the peptide itself appears to be a poor drug candidate due to its short plasma half-life (5 min), poor CNS permeability, and endocrine side effects [75]. Of note, ASMs such as CBZ [10,13], OXC, and VGB [10] have been described as possible triggers of SE in patients with AS; therefore, the discontinuation of such therapies may play a pivotal role to prevent or resolve the occurrence of SE.

Ultimately, infancy and childhood appear to be the most vulnerable periods for the occurrence of SE in AS: the youngest case described was 8 months old [19] and several authors observed a decreased incidence of SE with increasing age in these patients. This is consistent with the typical modifications in the epilepsy pattern these patients go through over time, leading to an increase in the proportion of patients that reach seizure freedom after puberty [12,18,76]. When considering long-term outcomes, understanding the clinical features of NCSE and providing adequate treatment are crucial steps in the care of patients with AS. Indeed, some authors hypothesize the appearance of repetitive, prolonged seizures, especially NCSE, with severe EEG abnormalities may coincide with the onset of developmental delay and with a transient or permanent decline in mental and motor functioning [19,28].

### 4.2. Status Epilepticus in Ring 20 Syndrome

No estimates about the incidence or prevalence of SE in patients with R20 could be gathered in the review: only case reports and small case series were highlighted from this extensive literature search, and large population studies are lacking. Once more, every attempt to evaluate the occurrence and features of SE in R20 may be hampered by the diagnostic challenge of identifying events with subtle clinical signs. Indeed, Vignoli et al. described R20′s distinct electro-clinical phenotype and already observed that SE in these patients mainly occurred without prominent motor features (namely, NCSE) [48]. Other than epilepsy, patients with R20 present with intellectual disability/developmental delay after seizure onset and behavioral features [77]: speech and executive abilities are frequently affected, resulting in apathy or hyperactivity, loss of social skills, obsessive behavior, psychosis, and autistic features [78]. Within this clinical picture, the occurrence of NCSE may be suspected whenever motor phenomena or changes in the behavioral, sleep, or seizure pattern occur, as stated for AS. In particular, mutism, aphasia, dulling of consciousness, loss of facial emotional expression and behavioral arrest with possible subtle motor features were reported as suggestive features of NCSE in these patients (Table 5); a peculiar sign pointing to NCSE may be the occurrence of spontaneous speech (one or two words) immediately after the resolution of ictal aphasia [78]. NCSE episodes recur over time in patients with R20; psychogenic stressors, such as concentration, fatigue, and emotional stress, turned out to be common triggers, while no fever-induced NCSE was reported [34,48,51]. Inoue et al. describe video games as NCSE facilitators in one patient in their sample; however, it is not clear whether this effect would be mediated through the same mechanism of other psychogenic stressors or by photic stimulation. Moreover, it remains uncertain whether psychogenic stressors may induce NCSE through hyperventilation, which has been reported as a trigger itself.

Data about SE treatment in R20 are consistent with international guidelines; no specific, targeted therapies are described in the literature. Only in two cases of 93, NCSE resolved after the administration of monotherapy with BZDs (intravenous DZP and CZP). The sporadic use of several ASMs is reported (Table 4). The impact of diagnostic delay on treatment responsiveness has not been systematically evaluated in patients with R20.

The outcome of recurrent NCSE in R20 patients is not clearly defined: some studies reported minimal psychosocial and cognitive impacts from NCSE, with a surprisingly benign course despite continuous discharges occurring over days [38]. On the other hand, a worse clinical course was described due to prolonged refractory epilepsy with NCSE by other authors, with patients developing cognitive deterioration, aggravation of learning disabilities, and behavioral problems until the progression of motor and verbal impairment [40,46]. De Falco et al. matched the clinical deterioration to a progressive disorganization of EEG background activity with increasing epileptic paroxysms [40].

Ultimately, it is not possible to define a common clinical outcome out of published reports; more evidence is needed to focus on the evolution of R20 syndrome with SE. However, available data suggest NCSE tends to persist into adulthood in these patients.

### 4.3. Status Epilepticus in Wolf–Hirschhorn Syndrome

Since large population studies about the incidence and prevalence of SE in WHS are not currently available, no conclusion can be drawn. Several authors describe SE occurring with a frequency ranging from 40% to 70% of cases [54,58,59]. SE with prominent motor features appeared to be the most frequent in WHS, with prevalent unilateral or generalized clonic or tonic–clonic semiology, during the first years of life [20,54,55,58]. Before the age of 5, early onset of seizure and worsening of electroclinical history often lead to SE [54]; then, the frequency of seizures and SE episodes gradually decreases after the age of 5 [54,58]. However, permanent motor impairment and a fatal outcome due to SE have been described during infancy [54,57,60].

Data about SE treatment in WHS are consistent with international guidelines; no specific, targeted therapies are described in the literature. Of note, Kagitani et al. observed SE was prevented dramatically in all cases treated with the administration of bromide, and SE occurring during infancy in WHS sometimes resulted in permanent disability or even death. Therefore, the authors suggest early administration of bromide (30–50 mg/kg/day) may be considered in order to prevent the occurrence of SE and related outcomes in WHS [54,60]. Unfortunately, no clear-cut predictive factors of adverse outcomes have been found in the present research. Shimizu et al. observed patients carrying intermediate (6–15 Mb) or large (>15 Mb) deletions of the short arm of chromosome 4 developed SE in 87% of cases, while those carrying small deletions (<6 Mb) had later onset of seizures and lower occurrence of SE (17%). Therefore, it has been hypothesized that the severity of epilepsy and SE might be related to different genetic substrates [59]. On the other hand, Battaglia et al. did not report any correlation between the deletion size and seizure type, severity, or onset age [58]. Ultimately, further studies about the genetic etiology and consequent pathophysiology of SE, explaining possible phenotypic–genotypic relationships, are needed.

### 4.4. Status Epilepticus in Down Syndrome

MSE was described in three patients, and no conclusions about specific features of SE in DS can be drawn from the present review since only two studies were included. MSE resolution with VPA was reported. SE occurrence in adults was reported (range: 29 to 59 years of age); anyway, no typical age at onset could be recognized. The only pediatric onset SE occurred during measles infection [61,62]. No specific ictal EEG pattern for SE is available.

### 4.5. Status Epilepticus in Ring 14 Syndrome

No specific SE features could be gathered from the description of 13 patients with SE and R14. Giovannini et al. suggested three stages of disease for patients with epilepsy and R14: (1) initial onset phase with frequent epileptic seizures and CSE in half of the cases; (2) a second stage (up to 20 years of age) characterized by NCSE and minor motor seizures; (3) an adult phase (>20 years) during which seizures tend to decrease till cessation [64]. Further evidence is needed in this field.

Zollino et al. [79] described 27 patients with R14 and highlighted intellectual disability in every patient except one, with occasional behavioral disorders (hyperactivity, burst of aggressiveness, and stereotypies): as suggested for other syndromes, the occurrence of NCSE may be suspected whenever clinicians or caregivers observe abrupt changes in the behavioral pattern of these patients.

### 4.6. Other CDAE

The literature research highlighted few studies regarding the occurrence of SE in the remaining chromosomal syndromes and the outcomes of interest were not extensively reported.

Two studies described the occurrence of ESES in Del15q13.3 patients [65,66]. Kevelam et al. hypothesize that the involvement of CHRNA7 in the microdeletion is likely responsible for the occurrence of ESES, due to its relationship with other forms of sleep-related epilepsy. No data about SE duration, frequency, and outcome have been reported in the literature.

The phenotype of Del18q syndrome is characterized by intellectual impairment, short stature, hypotonia, hearing loss, and dysmorphic features. Epilepsy is rare and in the literature, only one patient has been reported to develop CSE over a history of generalized tonic–clonic epilepsy; no treatment of epilepsy or SE was reported [67].

Del 1p36 syndrome is characterized by global intellectual impairment, characteristic craniofacial dysmorphism, congenital heart defects, obesity, precocious puberty, and hypotonia. Epilepsy occurs in 50–60% of cases and it is not reported to be drug-resistant. SE does not seem to be a hallmark of this syndrome as it was reported in only four patients in the literature [68]. More detailed reports of SE episodes in Del1p36 syndrome are needed to better characterize the frequency and the semiology of SE in these patients.

InvDup15 (or Idic15) syndrome clinical features include hypotonia and motor delays, intellectual disability, autism spectrum disorder, and early-onset, drug-resistant epilepsy. No specific SE phenotype was found among these patients.

The only patient with SE in Klinefelter syndrome also had interstitial Xp22.31 deletions, which were considered responsible for the severe phenotype. Furthermore, other suspected genetic alterations were not ruled out due to healthcare issues. To date, no episodes of SE have been reported in Klinefelter syndrome, Miller–Dieker syndrome, Pallister–Killian syndrome, and trisomy 12p.

Three studies about SE in R17 syndrome were included as secondary findings. R17 syndrome may include the deletion of the Miller–Dieker critical region (MDCR), which maps to the short arm of chromosome 17 at 17p13. The phenotype is milder if MDCR is not involved in R17 formation; however, four cases developed recurrent episodes of NCSE. Treatment with VPA and LTG helped reduce seizure frequency but did not stop NCSE in one case [72], while another responded to BZD [73].

## 5. Conclusions

The present review gathers the available evidence about SE clinical features, treatment, and outcomes among patients carrying CDAE. Table 8 summarizes the gathered evidence in the field. The vast majority of the included studies are case reports or small case series and regard AS and R20. Great heterogeneity in reporting the outcomes of interest emerges from the literature research; therefore, no proper inference can be made from the collected data. 

NCSE is frequently observed in AS and R20 and it represents a diagnostic challenge for caregivers and clinicians; abrupt changes in these patients’ typical behavior may represent clinical clues of NCSE and may require EEG recordings to confirm the diagnosis. Among patients with WHS, CSE was more frequently observed.

No specific, targeted therapies for SE in CDAE are available to date, and international guidelines on the management of SE should be followed; anecdotal reports about SE treatment with ASMs or other drugs are described in the text.

SE incidence gradually decreases with age over infancy in AS and WHS; no clear evidence about sex-related differences emerged from the present review. Other CDAE’s brief- and long-term outcomes are reported in the paper; however, no predictive factors of SE outcome could be identified. 

Further studies are needed to precisely characterize the clinical features, treatment options, and outcomes of SE in these patients.

## Figures and Tables

**Table 1:** Chromosomal disorders associated with epilepsy according to the ILAE.

Chromosomal Disorders and Epilepsy
15q13.3 Microdeletion Syndrome (Del15q13.3)
18q- Syndrome (Del18q)
InvDup (15) or IDIC (15)
Del 1p36
Angelman Syndrome (AS)
Down Syndrome (DS—Trisomy 21)
Klinefelter Syndrome (XXY)
Miller Dieker Syndrome (Del 17p)
Pallister Killian Syndrome (Tetrasomy 12p)
Ring 14 (R14) Syndrome
Ring 20 (R20) Syndrome
Trisomy 12p
Wolf-Hirschhorn Syndrome (WHS—Del 4p)

**Table 2:** Status Epilepticus in Angelman Syndrome.

Authors	Population (Number, Sex, Age)	Patients with SE (Number, Sex, Age)	Previous Epilepsy	SE Semeiology	SE Recurrence	SE Treatment
Sugimoto T. et al., 1992 [11]	N:3, M:2; 1–6 yo	N:1; M; 6 yo	Yes	NCSE	-	CZP
Matsumoto A. et al., 1992 [12]	N:8; Na; Na	N: 2; Na; 8–10 yo	Yes	NCSE	Yes	1: DZP; DZP and TRH (1 mg/day) 2: DZP;
Viani F. et al., 1995 [13]	N:18; M:12, F:6; 8 m–27 yo	N: 9; Na; < 6 yo	-	MSE	-	DZP, VPA, CLB
Rubin D.I. et al., 1997 [14]	N:3; M:3; 2 y 3 m	N: 1; Na; Na	Yes	NCSE (ESES)	Yes	-
Laan L.A. et al., 1997 [15]	N: 36; M: 20, F:16; 1–32 yo	N: 7; Na; Na,	Yes (n:5) No (n: 2)	NCSE, CSE, MSE	Yes	-
Galván-Manso M. et al., 2005 [16]	N:37; F:19, M:18; 3–23 yo	N: 15; Na; Na	-	NCSE	-	-
Espay A.J. et al., 2005 [17]	N:1; M; 29 yo	N: 1; Na; Na	Yes	NCSE	Yes	-
Uemura N. et al., 2005 [18]	N:23; M:10, F:13; 3 m–37 yo	N: 11; Na; 1–15 yo	Yes (n: 10), No (n: 1)	NCSE, CSE	Yes	-
Ohtsuka Y. et al., 2005 [19]	N:11; M:6, F:5; 3–15 yo	N: 10; M: 5, F: 5; 8 m–8 yo	-	NCSE	Yes	1: iv DZP, VPA; 3: iv DZP, CZP, CLB, VPA; 4: iv DZP, VPA; 5: iv DZP, VPA, ESM, ACTH; 6: iv DZP, CZP, VPA, ESM, ACTH; 7: iv DZP, CZP, VPA; 8: VPA; 9: VPA; 10: iv DZP, VPA; 11: CZP, VPA
Valente K.D. et al., 2006 [10]	N:19; Na; Na	N: 16; Na; Na	Yes	NCSE, MSE	Yes	-
Caraballo R.H. et al., 2007 [20]	N:15; Na; Na	N:17; Na; <1 yo	-	MSE	-	-
Yang X.Y. et al., 2010 [21]	N:14; M:4, F:10; 8 m–3 yo	N: 10; Na; Na	Yes (n: 9), No (n:1)	NCSE	Yes	-
Weber P., 2010 [22]	N:1; M:1; 7 yo	N: 1; M:1; 7 yo	Yes	CSE, NCSE	Yes	LEV
Valente K.D. et al., 2012 [23]	N:16; Na; Na	N:5; Na; Na	-	-	-	-
Nicita F. et al., 2015 [24]	N:1; M:1; Na	N:1; Na; Na	No	MSE	Yes	MDZ (two boluses), VPA, VGB, LEV was finally effective
Grocott O.R. et al., 2017 [25]	N:23; M:15, F:8; 2–31 yo;	N:3; Na; Na	-	NCSE	Yes	-
Worden L. et al., 2018 [26]	N:104; Na; <18 yo	N:13; M:9, F:4; 15 m–12 yo	Yes (n: 10), No (n: 3)	NCSE	Yes	Oral DZP
Pollack S.F. et al., 2018 [27]	N:185; M:102, F:83; 1–44 yo	N: 1; Na; Na		MSE	-	-
Bindels-de Heus K.G.C.B. et al., 2020 [28]	N:100; M:50, F:50; <18 y	N: 24; Na; Na		NCSE, CSE	Yes	-
Melikishvili G et al., 2022 [29]	N:2; Na; Na	N:2; Na; Na		NCSE	Yes	1: DZP, VPA, B6; KD 4:1 radio;2: KD

NCSE: Non-convulsive Status Epilepticus; CSE: Convulsive Status Epilepticus; MSE: Myoclonic Status Epilepticus; ESES: electrical status epilepticus during slow-wave sleep; CZP: clonazepam; DZP: diazepam; TRH: thyrotropin-releasing hormone; VPA: valproate; CLB: clobazam; ESM: ethosuximide; ACTH: adrenocorticotropic hormone; LEV: levetiracetam; MDZ: midazolam; VGB: vigabatrin; KD: ketogenic diet.

**Table 3:** Clinical features of Non-Convulsive Status Epilepticus in patients with Angelman Syndrome.

Positive Findings	Negative Findings
Motor phenomena: clumsiness, drooling of saliva, eye deviation, eye dropping Minor motor components: blinking, trembling, and clusters of jerky movements (especially in the distal regions of the limbs) Behavioral changes: agitation, emotional liability Sleep: somnolence Seizures: increased seizure frequency	Motor phenomena: psychomotor arrest (staring), arrest of stereotyped movements (i.e.: hand flapping) Behavioral changes: Minor readiness to laugh, loss of interest in food Seizures: lower frequency of motor seizures in those regularly experiencing them Sleep: poor quality Consciousness: decreased alertness and responsiveness, poor communication, loss of acquired milestones

**Table 4:** Status Epilepticus in Ring 20 Syndrome.

Authors	Population (Number, Sex, Age)	Patients with SE (Number, Sex, Age)	Previous Epilepsy	SE Semeiology	SE Recurrence	SE Treatment
Inoue Y. et al., 1997 [32]	N:6; F:4, M:2; 13–48 yo	N:6; F:4, M:2; 3–14 yo	No (N: 5); Yes (N: 1)	NCSE	Yes (daily)	1: iv DZP; 2: iv lidocaine; 3-4-5-6,
Kobayashi K. et al., 1998 [33]	N:2; F:1, M:1; 22 yo	N:2; F:1, M:1; 22	Yes	CSE	Yes	1: PB; 2: Na
Petit J. et al., 1999 [34]	N:3; M:1, F:2; 22–43 yo;	N:3; M:1, F:2; Na	Yes	NCSE	Yes	-
Augustijn P. et al., 2001 [35]	N:4; F:3, M:1; 8–13 yo;	N:4; F:3; M:1; 8–14 yo	Yes	NCSE	Yes	-
Gomes M. et al., 2002 [36]	N:1; M:1; 12 yo	N:1; M:1, 11.5	Yes	NCSE	Yes	-
Gonzalez-Delgado M. et al., 2004 [37]	N:1; Na; 18 yo	N:1; Na; Na	Yes	NCSE	-	CZP
Locharernkul C. et al., 2005 [38]	N:2; F:2; 25–37 yo	N:2; F:2; 21–27 yo	Yes	NCSE	-	1: iv PHT, iv VPA; 2: iv VPA
Alpman A. et al., 2005 [39]	N:1; M:1; 14 yo	N:1; M:1; 11 yo	Yes	NCSE	Yes	-
de Falco F. et al., 2006 [40]	N:1; F:1; 46 yo	N:1 F;1; 39	Yes	CSE + NCSE	Yes	-
Zou Y.S. et al., 2006 [41]	N:1; F:1; 26 yo	N:1; F:1; Na	Yes	NCSE	Yes	-
Elghezal H. et al., 2007 [42]	N:1; F:1; 12 yo	N:1; F:1; Na	Yes	NCSE	Yes	-
Jacobs J. et al., 2009 [43]	N:1; M:1; 13 yo	N:1; M:1; Na	Yes	NCSE	Yes	-
Elens I. et al., 2012 [44]	N:6; M:4, F:2; 4–53 yo	N:6; M:4, F:2; 4–54 yo	Yes	NCSE	-	-
Radhakrishnan A. et al., 2012 [45]	N:3; M:3; 10–20 yo	N:2; M:2; 10	Yes	NCSE	-	-
Wechapinan T. et al., 2014 [46]	N:1; F:1; 11 yo	N:1; F:1; 11 yo	Yes	NCSE	-	iv MDZ, LTG, LEV, CBZ, ZNS
Onder H. et al., 2015 [47]	N:1; F:1; 25 yo	N:1; F:1, Na	Yes	NCSE	-	LCS
Vignoli A. et al., 2016 [48]	N:25; F:17, M:8; 8–59 yo	N:22; Na; Na	Yes	NCSE	Yes	-
Hirano Y. et al., 2016 [49]	N:1; F:1; 17 yo	N:1, F:1, 13 yo	Yes	CSE	Yes	MDZ, PHT, PB, sodium thiopental
de Moura F. et al., 2016 [30]	N:24; F:10, M:14; 17–57 yo	N:24; F:10, M:14; Na	-	-	-	-
Gago-Veiga A.B. et al., 2018 [31]	N:6; F:5, M:1; 11–30 yo	N:6; F:5, M:1; 2–15 yo	Yes	NCSE (N:6); CSE (N:1)	Yes	-
Lee H. et al., 2020 [50]	N:1; F:1; 17 yo	N:1; F:1; 8 yo	Yes	NCSE	-	-
Yamagishi H. et al., 2020 [51]	N:2; F:1, M:1, 6–11 yo	N:2; F:1, M:1, Na	Yes	NCSE	-	LCS
Kishore V.K. et al., 2022 [52]	N:1; Na; Na;	N:1; Na; Na	Yes	NCSE	-	Iv mPRED
Borkovic M. et al., 2022 [53]	N:4; F:3, M:1; 9–18 yo	N:2; F:2; Na	-	NCSE + CSE	-	-

NCSE: Non-convulsive Status Epilepticus; CSE: Convulsive Status Epilepticus; DZP: diazepam; PB: phenobarbital; CZP: clonazepam; PHT: phenytoin; VPA: valproic acid; MDZ: midazolam; LTG: lamotrigine; LEV: levetiracetam; CBZ: carbamazepine; ZNS: zonisamide; LCS: lacosamide; mPRED: methylprednisolone.

**Table 5:** Clinical features of Non-Convulsive Status Epilepticus in patients with Ring 20.

Positive Findings	Negative Findings
Consciousness: confusion Motor phenomena: wandering Minor motor components: automatisms, jerks, myoclonias in the distal regions of the limbs Seizures: mild clonic or tonic seizures over a prevalent confusional state Behavioral changes: agitation, fear, emotional liability, screaming Speech: Sudden post-aphasic speech	Consciousness: impaired vigilance Behavioral changes: Loss of emotional facial expressions, unresponsiveness, or mental slowing; behavioral arrest Speech: Diminished spontaneous speech, aphasia, mutism.

**Table 6:** Status Epilepticus in other Chromosomal Disorders Associated with Epilepsy.

Syndrome	Authors	Patients with the Syndrome (Number, Sex, Age)	Patients with SE (Number, Sex, Age)	Previous Epilepsy	SE Semeiology	SE Recurrence	SE Treatment
WHS	Kanazawa O. et al., 1991 [56]	N:2; M:1, F:1; 2–3 yo	N:1; F:1; 2 yo	No	CSE, Hemiclonic SE	Yes	DZP, PHT, ZNS
WHS	Kagitani K. et al, 2005 [54]	N: 11; F:8; M:3; 2–25 yo	N:11; F:8; M:3; 2–3 yo	Yes	CSE, Hemiclonic SE, NCSE	Yes	DZP (N: 8); LD (N:1); TS (N:1); TP (N:1); Bromide (N:4)
WHS	Mitić V. et al., 2011 [57]	N:1; F:1; 9 m	N:1; F:1; 9 m	No	Hemiclonic SE	Yes	PB
WHS	Caraballo R.H. et al., 2007 [20]	N:2; Na; Na	N:2; Na; Na	Na	MSE	Yes	-
WHS	Battaglia A. et al., 2009 [58]	N:87; F:54; M:33; 1–25.6 yo	N:36; Na; <3y yo	Yes	CSE	-	-
WHS	Valente K.D. et al., 2003 [55]	N:1; F:1; 2 yo	N:1; F:1; 2 yo	Yes	NCSE, MSE	Yes	PB
WHS	Shimizu K. et al., 2014 [59]	N: 22; F: 18, M: 4; Na	N: 14; F:11; M:3; Na	Yes	-	-	Bromide (N:4)
WHS	Itakura A. et al., 2016 [60]	N:1; F:1; 3 m	N:1; F:1; 3 m	No	Hemiclonic SE, CSE	-	MDZ; PB
DS	Vignoli A. et al., 2011 [61]	N:22; F:11, M:11; 28–64 yo	N:3; M:2, F:1; Na	Yes	MSE	Yes	VPA
DS	Takasugi H. et al., 2000 [62]	N:1; Na; Na	N: 1; Na; Na	No	-	-	-
R14	Giovannini S. et al., 2010 [63]	N:1; M:1; 9 yo	N:1; M:1; 15 m	No	CSE; NCSE	-	BZD, PHT
R14	Giovannini S. et al., 2013 [64]	N:22; F:9, M:13; 2–22 yo	N:13; F:3, M:10; Na	Yes (N:5); No (N:3)	CSE (N:9); NCSE (N:3); Na (N:3)	-	BZD, PHT
Del15q13.3	Whitney R. et al., 2021 [65]	N:13; F:9, M:4; 3–15 yo	N:3; F:2, M:1; 3–8.5 yo	-	NCSE (N:2, ESES (N:1)); CSE (N:1);	-	-
Del15q13.3	Kevelam S.H. et al., 2012 [66]	N:13; Na; Na	N:1; Na; 7 yo	No	NCSE (ESES)	-	-
Del18q	Kanazawa O. et al., 1989 [67]	N:1; M:1; 15 yo	N:1; M:1; 15 yo	Yes	Hemiclonic SE	-	-
Del1p36	Kanabar G. et al., 2012 [68]	N:4; M:3, F:1; 2–16 yo	N:4; M:3, F:1; 2–16 yo	Yes	-	-	-
InvDup15	Friedman D. et al., 2016 [69]	N:19; F:8, M:11; 2.5–39 yo	N:7; F:3, M:4; 10–26 yo	Yes	-	-	-
XXY	Brault J. et al., 2021 [70]	N:1; M:1,7 m	N:1; M:1; 7 m	No	-	-	BZD, PB, PHT, LEV
R17	Ricard-Mousnier B. et al., 2007 [71]	N:1; M:1; 4 yo	N:1; M:1; 4 yo	Yes	NCSE	-	-
R17	de Palma L. et al., 2015 [72]	N:1, F:1, 17 yo	N:1; F:1; 11 yo	Yes	NCSE	-	-
R17	Coppola A. et al., 2017 [73]	N:1, F:1, 31 yo	N: 1; F: 1; 28 yo	Yes	NCSE	-	BDZ

NCSE: Non-convulsive Status Epilepticus; CSE: Convulsive Status Epilepticus; MSE: Myoclonic Status Epilepticus; ESES: electrical status epilepticus during slow-wave sleep; DZP: diazepam; PHT: phenytoin; ZNS: zonisamide; PB: phenobarbital; MDZ: midazolam; BZD: benzodiazepine; VPA: valproic acid; LEV: levetiracetam; LD: lidocaine; TS: thiamylal sodium; TP: thiopental.

**Table 7:** Status Epilepticus semiology in patients with Wolf-Hirschhorn Syndrome.

Semiology	Number of Patients
Generalized tonic–clonic	4 [54]
Tonic	2 [54]
Myoclonic	2 [58]
Focal motor, Hemiclonic	7 [54], 4 [54,56,57,60]
Generalized or focal motor SE	36 [55,58]

**Table 8:** Synoptic table. Status Epilepticus in CDAE.

Syndrome	SE Semeiology	Patients with SE Reported (Number)	Age Range of SE	SE Recurrence	Ictal EEG	SE Treatment
AS	NCSE, MSE	150	8 m–15 yo	Yes	Diffuse high voltage 1.5–3 Hz SW	Remove triggers; BZD; VPA; LEV
R20	NCS	93	2–54 yo	Yes	Generalized or predominantly frontal slow waves and spikes or SW	BZD, LCS
WHS	CSE, Hemiclonic	67	3 m–3 yo	Yes	-	BZD, Bromide, PB
DS	MSE	4	-	-	-	VPA
R14	CSE	14	-	-	-	BZD, PHT
Del15q13.3	ESES	4	3–8.5 yo	-	SW during sleep	-
Del18q	Hemiclonic	1	15 yo	-	-	-
Del1p36	-	4	2–16 yo	-	-	-
InvDup15	-	7	10–26 yo	-	-	-
XXY	-	1	7 m	-	-	-
Ring 17	NCSE	3	4–28 yo	-	Sub-continuous diffuse slow SW, slow background activity.	BZD

SE: status epilepticus; NCSE: non-convulsive status epilepticus; MSE: myoclonic status epilepticus; CSE: convulsive status epilepticus; SW: spike and waves; BZD: benzodiazepines; VPA: valproate; LEV: levetiracetam; LCS: lacosamide; PB: phenobarbital; PHT: phenytoin.

## Data Availability

Not applicable.
